# Aging LGBT Adults’ Access to Social Resources According to LGBT Identity and Sociodemographics

**DOI:** 10.1093/geroni/igab046.657

**Published:** 2021-12-17

**Authors:** Krystal Kittle, Kathrin Boerner, Kyungmin Kim

**Affiliations:** 1 University of Nevada, Las Vegas, Las Vegas, Nevada, United States; 2 University of Massachusetts Boston, Boston, Massachusetts, United States; 3 Seoul National University, Seoul, Seoul-t'ukpyolsi, Republic of Korea

## Abstract

Research suggests that social resources positively influence the health and well-being of lesbian, gay, bisexual, and transgender (LGBT) aging adults, but their access to social resources may vary according to LGBT identity. Using data from Aging with Pride: National Health, Aging, and Sexuality/Gender Study (N=2,536), multivariate models tested how access to social resources varied by LGBT identity and whether the effect of LGBT identity showed additional variations by sociodemographic characteristics (i.e., age and education) among aging LGBT adults. Lesbian respondents had larger social networks than gay respondents, while gay respondents had smaller networks than transgender respondents. Lesbian respondents had more social support and community belonging than other identity groups. Bisexual male respondents and transgender respondents had less support than gay respondents and bisexual male respondents reported less community belonging than gay respondents. Education and age moderated the association between LGBT identity and social support. Findings highlight the importance of considering social support separately from social network size with the understanding that large social networks do not necessarily provide ample social support and this distinction was particularly relevant for transgender respondents who had larger social networks, but less social support than gay respondents. Results also suggest that feelings of LGBT community belonging vary among LGBT identity groups. Health and human service professionals should not only consider the sexual and gender identity of their aging LGBT clients, but also consider the clients’ additional sociodemographic characteristics when assessing their access to social resources.

